# Mechanisms of pressure-diuresis and pressure-natriuresis in Dahl salt-resistant and Dahl salt-sensitive rats

**DOI:** 10.1186/1472-6793-12-6

**Published:** 2012-05-14

**Authors:** Daniel A Beard, Muriel Mescam

**Affiliations:** 1 Center for Computational Medicine, Biotechnology and Bioengineering Center, Department of Physiology, Medical College of Wisconsin, Milwaukee, WI, USA

## Abstract

**Background:**

Data on blood flow regulation, renal filtration, and urine output in salt-sensitive Dahl S rats fed on high-salt (hypertensive) and low-salt (prehypertensive) diets and salt-resistant Dahl R rats fed on high-salt diets were analyzed using a mathematical model of renal blood flow regulation, glomerular filtration, and solute transport in a nephron.

**Results:**

The mechanism of pressure-diuresis and pressure-natriuresis that emerges from simulation of the integrated systems is that relatively small increases in glomerular filtration that follow from increases in renal arterial pressure cause relatively large increases in urine and sodium output. Furthermore, analysis reveals the minimal differences between the experimental cases necessary to explain the observed data. It is determined that differences in renal afferent and efferent arterial resistances are able to explain all of the qualitative differences in observed flows, filtration rates, and glomerular pressure as well as the differences in the pressure-natriuresis and pressure-diuresis relationships in the three groups. The model is able to satisfactorily explain data from all three groups without varying parameters associated with glomerular filtration or solute transport in the nephron component of the model.

**Conclusions:**

Thus the differences between the experimental groups are explained solely in terms of difference in blood flow regulation. This finding is consistent with the hypothesis that, if a shift in the pressure-natriuresis relationship is the primary cause of elevated arterial pressure in the Dahl S rat, then alternation in how renal afferent and efferent arterial resistances are regulated represents the primary cause of chronic hypertension in the Dahl S rat.

## Background

Animal models of salt- and/or angiotensin II-induced chronic hypertension have revealed shifts in the observed pressure-natriuresis and pressure-diuresis relationships to higher pressures, as well as altered renal blood flow regulation [1-6]. The salt-sensitive Dahl S (SS) rat is a widely studied example of an animal that develops hypertension, associated with a shift of the pressure-natriuresis relationship (relationship between sodium excretion and arterial pressure) to higher pressures, when fed a high-salt diet. When maintained on high salt (e.g., 8% NaCl in chow) the kidneys of these animals are found to excrete a given amount of sodium per unit time at a higher input arterial pressure than the kidneys of control animals fed low-salt diets and of strains, such as the salt-resistant Dahl R (SR) rat, that do not exhibit salt-induced hypertension. Thus sodium balance (dietary sodium input minus sodium excretion) is achieved in hypertensive animals at higher pressures than in normotensive animals [7].

Guyton and Coleman and coworkers hypothesize that a shift in the pressure-natriuresis relationship to higher pressures is one of the central causal mechanisms of chronic hypertension in salt-sensitive hypertension [8]. Other investigators suggest that angiotensin II- and salt-induced increases in sympathetic nervous activity in the vasculature may be a primary causal factor in salt-sensitive hypertension while the shift in the renal pressure-natriuresis relationship may not [9-11]. Not only is it unclear whether and when the observed changes in the pressure-natriuresis relationship are causes or consequences of chronic hypertension (or in some way both), it remains unclear what specific aspects of renal physiology are altered in salt-sensitive hypertension, underlying the observed changes in the pressure-natriuresis and pressure-diuresis relationships.

Here we analyze data on blood flow regulation, renal filtration, and urine output in SS rats fed on high-salt (hypertensive) and low-salt (prehypertensive) diets and salt-resistant SR rats fed on high-salt diets. We use a simple mathematical model of renal blood flow regulation, glomerular filtration, and solute transport in a nephron to reveal the minimal differences between the three cases necessary to explain the observed data. It is found that the differences in renal blood flow, glomerular filtration, and pressure-diuresis and pressure-natriuresis relationships may be explained based solely on differences in afferent and efferent arteriole regulation in the hypertensive (high-salt) SS compared to the salt-resistant SR and the low-salt SS controls.

### Sources of data

Data from the SS and SR rats used for model identification are obtained from Roman [12]. Additional independent data from SS and SR rats for model comparison were obtained from Roman and Kaldunski [13]. For these data sets measurements were made in denervated kidneys perfused *in vivo* with plasma levels of vasopressin, aldosterone, corticosterone, and norepinephrine clamped. Data from three experimental groups are analyzed: high-salt fed hypertensive SS rats with baseline pressure of 158 ± 2 mmHg, low-salt fed prehypertensive SS rats with baseline pressure of 133 ± 1 mmHg, and high-salt fed SR rats with baseline pressure of 124 ± 1 mmHg. Additional data for comparison to model predictions are obtained from Thompson and Pitts [14] and were obtained in normal dogs in which glomerular filtration rate was modulated by varying renal arterial pressure. (Data from Thompson and Pitts on adrenalectomized and sympathectomized dogs show similar trends.)

## Methods

**Figure 1:**
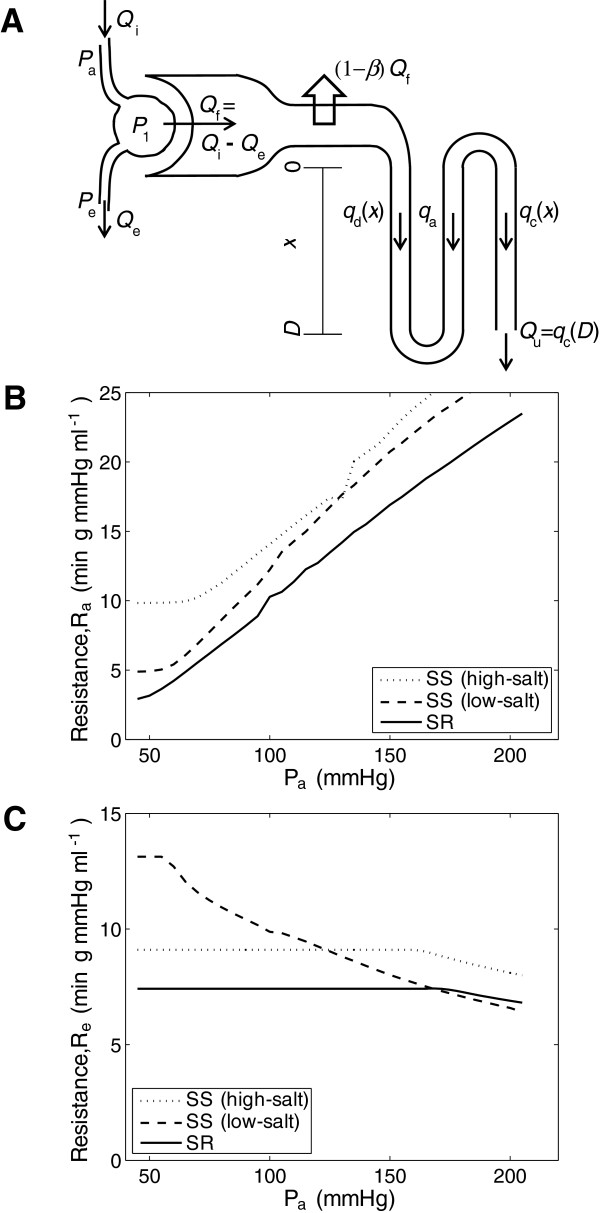
**A. Diagram of model. B**. Afferent arterial resistances governed by Equation (8), with parameter values from Table 1, for the three experimental cases analyzed. **C**. Efferent arterial conductivities governed by Equations (6), with parameter values from Table 1, for the three experimental cases analyzed.

The mathematical model of renal blood flow, glomerular filtration, and mass transport in nephrons (diagrammed in Figure 1) is composed of two main components, a model for renal blood flow and glomerular filtration and a model for mass transport in a representative nephron. Both components are based on modifications made to models presented in Chapter 20 of Keener and Sneyd [15]. The blood flow and filtration model predicts glomerular filtration rate, glomerular pressure, and renal blood flow as functions of input arterial pressure. The predicted glomerular filtration rate and pressure serve as inputs to the nephron model, which predicts concentrations of sodium and flows in the descending and ascending limbs of the loop of Henle and a combined intersitium/ascending vasa recta space. Predictions of the overall model are compared to data on renal blood flow, glomerular filtration, glomerular pressure, efferent capillary pressure, urine flow, and sodium excretion in low-salt fed prehypertensive and high-salt fed hypertensive SS and high-salt fed SR rats.

**Table 1:** Adjustable parameter values

		**Dahl-R**	**Dahl-S**	
		**high-salt**	**low-salt**	**high-salt**
glomerular hydraulic permeability times length	*K*_*f*_*L* (ml·min^−1^·g^−1^·mmHg^−1^)	0.0886	a	a
resistance associated with distal tubule	*R*_*d*_ (min·g·mmHg·ml^−1^)	7.4959	a	a
afferent arteriole resistance parameter	*R*_*a*_^0^ (ml·min^−1^·g^−1^·mmHg^−1^)	37.7673	a	a
minimum afferent arteriole resistance	*R*_*a*_^min^ (ml·min^−1^·g^−1^·mmHg^−1^)	2.8758	4.88	9.84
afferent autoregulation parameter	*Q*_*i0*_ (ml·min^−1^·g^−1^)	5.5796	4.28	b
afferent autoregulation parameter	*n*_*i*_	9.5614	a	a
maximum efferent arteriole resistance	*R*_*e*_^max^ (ml·min^−1^·g^−1^·mmHg^−1^)	7.4185	13.1	9.10
efferent arteriole resistance parameter	*b* (ml·min^−1^·g^−1^·mmHg^−1^)	37.5995	a	a
efferent arteriole resistance parameter	*m* (ml·min^−1^·g^−1^·mmHg^−2^)	0.5269	0.577	0.54
TGF concentration parameter	*C*_*TGF*_ (mM)	25.0	a	a
efferent capillary pressure fitting parameter	*P*_*e0*_ (mmHg)	10.4	a	8.32
efferent capillary pressure fitting parameter	*P*_*e1*_ (mmHg)	15.1	a	a
efferent capillary pressure fitting parameter	*P*_*e2*_ (mmHg)	136.5	a	a
efferent capillary pressure fitting parameter	*n*_*Pe*_ (unitless)	5.93	a	a
sodium permeability of the descending limb	*H*_*d*_ (ml·min^−1^·g^−1^·mm^−1^)	7.70 × 10^−3^	a	a
hydraulic permeability of the descending limb	*K*_*d*_ (ml·min^−1^·g^−1^·mmHg^−1^·mm^−1^)	8.3889 × 10^−4^	a	a
hydraulic permeability of the collecting duct	*K*_*c*_ (ml·min^−1^·g^−1^·mmHg^−1^·mm^−1^)	1.8777 × 10^−5^	a	a
maximum sodium reabsorption rate in ascending limb	*P*_*max*_ (ml·mM·min^−1^·mm^−1^·g^−1^)	29.172	a	a
apparent Michaelis-Menten constant for sodium reabsorption	*K*_*m*_ (mM)	50.933	a	a

(a) value same as Dahl-R value.

(b) value same as Dahl-S value.

While the treatment of a single nephron as representative of whole kidney is a gross simplification compared to models that capture heterogeneities in loop length and the three-dimensional architecture of the tubules and vasculature [16-19], the model developed here is appropriate to capture the physiological function analyzed here. Models of renal flow regulation and tubuloglomerular feedback [20-22], transport in the proximal tubule and cortex [23-25], medulla [17,18,26-35], collecting duct [34,36], and other components [16] have been developed to capture much more biophysical detail than the whole-kidney model developed here. Yet we are aware that no previously developed model, however simplified, combines renal hemodynamics, filtration, and tubular transport to simulate and analyze data on whole-kidney pressure-natriuresis function.

Previous models of renal system that capture overall kidney function, including the pressure-natriuresis and pressure- diuresis phenomena, have been developed [37-40]. However, these models do not capture spatially distributed transport in kidney, even at the simplified level of the model developed here.

### **Governing equations for blood flow and filtration**

Flow and filtration along glomerular capillaries is governed by the conservation equation for flow in a glomerular capillary, *q*_1_(*y*):

(1) $$\frac{dq_{1}}{dy}=K_{f}(P_{2}-P_{1}+\Pi_{c}),y\in (0,L)\text{,}$$

where *y* is the distance along the glomerular capillary, *K*_*f*_ is the hydraulic permeability, Π_*c*_(*y*) is the oncotic pressure in the plasma, and *P*_1_ and *P*_2_ are hydrostatic pressures in the capillary and Bowman’s capsule, respectively. This expression assumes that the rate of fluid loss from the capillary is linearly proportional to the pressure difference driving force and that pressure remains effectively constant along the length of the glomerular capillary. Blood enters the capillary at *y* = 0 with an input oncotic pressure Π_*c*_(*y* = 0) = Π_*i*_ = 28 mmHg and input flow *q*_1_(*y* = 0) = Q_*i*_. Assuming a linear relationship between concentration and oncotic pressure, we have

(2) $$\Pi_{c}(y)q_{1}(y)=\Pi_{i}Q_{i}\text{.}$$

Combining Equations (1) and (2) yields

(3) $$\frac{dq_{1}}{dy}=K_{f}\left(P_{2}-P_{1}+\Pi_{i}\frac{Q_{i}}{q_{1}}\right)\text{,}$$

which is a separable equation that can be solved to yield the following relationship between input flow *q*_1_(*y* = 0) = *Q*_*i*_ and output flow *q*_1_(*y* = *L*) = *Q*_*e*_:

(4) $$\frac{Q_{e}}{Q_{i}}+\alpha \ln\left(\frac{Q_{e}/Q_{i}-\alpha }{1-\alpha }\right)=1-\frac{K_{f}L\Delta P}{Q_{i}}\text{,}$$

where *ΔP* = *P*_1_ − *P*_2_, and *α* = *Π*_*i*_/*ΔP*. The filtrate flow (glomerular filtration rate) is computed from the difference between input and output blood flows, *Q*_*f*_ = *Q*_*i*_ − *Q*_*e*_.

Blood flow into the glomerulus satisfies the Ohm’s Law relationship

(5) $$P_{a}-P_{1}=Q_{i}\cdot R_{a}\text{,}$$

where *P*_*a*_ is the input arterial pressure and *R*_*a*_ is the afferent arterial resistance, which is phenomenologically modeled using the following increasing function of filtration

(6) $$R_{a}(Q_{i})=R_{a}^{o}\frac{Q_{i}{}^{n_{i}}}{Q_{i}{}^{n_{i}}+Q_{i0}{}^{n_{i}}}\cdot \frac{c_{a}(0)}{C_{\mathrm{TGF}}+c_{a}(0)}+R_{a}^{\min}\text{,}$$

where *R*_*a*_^*o*^, *R*_*a*_^min^,*n*_*i*_, *Q*_*a*0_, and *C*_*TGF*_ are adjustable parameters, and *c*_*a*_(0) is the sodium concentration in the ascending limb at the location where it feeds into the distal tubule. The factor $Q_{i}{}^{n_{i}}/(Q_{i}{}^{n_{i}}+Q_{i0}{}^{n_{i}})$ increases smoothly with increasing blood flow, representing an autoregulatory vasoconstriction. The factor *c*_*a*_(0)/(*C*_*TGF*_ + *c*_*a*_(0)) is employed to account for tubular-glomerular feedback: increasing salt concentration in the distal tubule stimulates vasoconstriction. (The concentration *c*_*a*_(0) is obtained from the transport component of the model, detailed below.)

Similarly blood flow out of the glomerulus satisfies the Ohm’s Law relationship

(7) $$P_{1}-P_{e}=Q_{e}\cdot R_{e}\text{,}$$

where *P*_*e*_ is the efferent capillary pressure and *R*_*e*_ is the efferent arterial resistance, which is phenomenologically modeled using the following decreasing function of *P*_*1*_, the input pressure into the arteriole.

(8) $$R_{e}(P_{1})=\{\begin{array}{l} R_{e}^{\max}\text{,}\;P_{1}\leq (b-R_{e}^{\max})/m\\ b-mP_{1}\text{,}\;P_{1}>(b-R_{e}^{\max})/m \end{array}$$

where *R*_*e*_^max^, *b*, and *m* are adjustable parameters. Thus the efferent arteriole is assumed to contribute to the decreasing behavior of the resistance in direct response to increases in pressure beyond a certain cutoff value of *P*_*1*_. Equations (6) and (8) predict that afferent resistance increases and efferent resistance decreases as renal perfusion pressure is increased, as illustrated in Figure 1. The tubular-glomerular feedback component of the model acts in the same direction as the autoregulatory factor in Equation (6). As pressure increases, filtration rate increases, leading to higher concentrations in the distal tubule, decreasing afferent conductivity.

Roman [12] reported measurements of pressures in peritubular capillaries (capillaries downstream of outer cortical glomeruli). As the model described here does not distinguish between corticomedullary and juxtamedullary glomeruli, the reported peritubular capillary pressures are compared to the model variable *P*_*e*_, efferent capillary pressure. Data on *P*_*e*_ as a function of arterial pressure are used to fit representative function for *P*_*e*_(*P*_*a*_):

(9) $$P_{e}=P_{e0}+P_{e1}\frac{P_{a}^{n_{\mathrm{pe}}}}{P_{a}^{n_{\mathrm{pe}}}+P_{e2}^{n_{\mathrm{pe}}}}\text{,}$$

which invokes four additional adjustable parameters, *P*_*e0*_, *P*_*e1*_, *P*_*e2*_, and *n*_*pe*_.

Filtrate flow satisfies the Ohm’s Law relationship

(10) $$P_{2}-P_{d}=R_{d}Q_{f}\text{,}$$

where *P*_*d*_ is the distal tubule pressure and *R*_*d*_ is the resistance associated with this pressure drop, assumed constant. In the absence of data on distal tubule hydrostatic pressure, we assume a simple linear proportionality between arterial pressure and *P*_*d*_:

(11) $$P_{d}=a_{\mathrm{Pd}}P_{a}\text{,}$$

where *a*_*Pd*_ is set to 0.02, which gives a value of distal tubule pressure of 2.0–3.6 mmHg over a range of renal perfusion pressure of 100 to 180 mmHg.

Equations (4), (5), (7), and (10), invoking 14 adjustable parameters (see Table 1), are solved for the four unknowns *Q*_*i*_, *Q*_*e*_, *P*_*1*_, and *P*_*2*_ to provide model predictions of these flows and pressures, as well as functions of input pressure *P*_*a*_.

### **Governing equations for nephron**

Mass transport in nephrons is represented using a one-dimensionally distributed model accounting for flows and concentrations in a single representative nephron. Thus three-dimensional interactions and the anatomical heterogeneity of loop lengths are not taken into account. Nevertheless, the model is able to effectively match observed pressure-diuresis and pressure-natriuresis relationships. The nephron model, diagramed in Figure 1, simulates flow and sodium concentration in four regions: the descending and ascending limbs of the loop of Henle, the collecting duct, and a combined ascending vasa recta/interstitium region. Fluid flows in these regions are denoted *q*_*d*_, *q*_*a*_, *q*_*c*_, and *q*_*s*_; sodium concentrations are denoted *c*_*d*_, *c*_*a*_, *c*_*c*_, and *c*_*s*_, where subscripts ‘d’, ‘a’, ‘c’, and ‘s’ indicate descending limb, ascending limb, collecting duct, and interstitial space. After passing through the proximal tubule, filtrate enters the descending limb at spatial position *x* = 0; the nephron region is defined over the spatial domain *x* ∈ [0, *D*], where *D* = 2 mm is the length of the segments of the nephron.

Fluid transport between the interstitium and the descending limb is assumed to be linearly proportional to the combined mechanical and osmotic pressure driving force, *P*_*d*_ + *Π*_*s*_ − *P*_*s*_ + 2*RT*(*c*_*d*_ − *c*_*s*_), where *P*_*d*_ is the hydrostatic pressure in the descending limb, *Π*_*s*_is the osmotic pressure in the interstitium, *P*_*s*_ is the hydrostatic pressure in the interstitium, and *c*_*d*_ and *c*_*s*_ are the Na^+^ concentrations in the descending limb and the interstitium. The factor of 2 multiplying the concentration gradient term arises because it is assumed that chloride concentration equals sodium concentration, and sodium plus chloride represent the major contributor to the osmotic gradient. With the hydraulic permeability constant *K*_*d*_ mass conservation yields the equation for *q*_*d*_, the flow in the descending limb:

(12) $$\frac{dq_{d}}{dx}=K_{d}(-\Delta P_{d}+2RT(c_{d}-c_{s})),x\in [0,D]$$

where *ΔP*_*d*_ = *P*_*d*_ + *Π*_*s*_ − *P*_*s*_, and interstitial osmotic and hydrostatic pressures are set to *Π*_*s*_ = 17 mmHg and *P*_*s*_ = 3 mmHg.

The ascending limb is assumed impermeable to water, and thus flow in the ascending limb, *q*_*a*_, is constant:

(13) $$\frac{dq_{a}}{dx}=0.$$

The governing equation for *q*_*c*_, the flow in the collecting duct is analogous to the equation for *q*_*d*_.

(14) $$\frac{dq_{c}}{dx}=K_{c}(-\Delta P_{c}+2RT(c_{c}-c_{s}))\text{,}$$

where *ΔP*_*c*_ = *P*_*c*_ + *Π*_*s*_ − *P*_*s*_ and *K*_*c*_ is the hydraulic permeability in the collecting duct. The hydrostatic pressure in the collecting duct is assumed to be 1 mmHg lower than that in the distal tubule: *P*_*c*_ = *P*_*d*_ − 1 mmHg.

Since total volume is conserved

(15) $$\frac{dq_{s}}{dx}=-\frac{d}{dx}(q_{d}+q_{a}+q_{c})\text{.}$$

Sodium transport is assumed to be governed by passive permeation in the descending limb and collecting duct and by active transport in the ascending limb. The governing equations for Na^+^ flux in descending limb is given by

(16) $$\frac{d(q_{d}c_{d})}{dx}=H_{d}(c_{s}-c_{d})\text{,}$$

where *H*_*d*_ is the descending limb permeability. The transport rate in the ascending limb is given by

(17) $$\frac{d(q_{a}c_{a})}{dx}=-P(c_{a})=-\frac{P_{\max}c_{a}}{c_{a}+K_{m}}\cdot \frac{{\left(C_{a,\max}\right)}^{5}}{{\left(c_{a}\right)}^{5}+{\left(C_{a,\max}\right)}^{5}}\text{,}$$

where the factor

$$\frac{P_{\max}c_{a}}{c_{a}+K_{m}}$$

models a saturable process, with *P*_*max*_ and *K*_*m*_ adjustable parameters. The factor

$$\frac{{\left(C_{a,\max}\right)}^{5}}{{\left(c_{a}\right)}^{5}+{\left(C_{a,\max}\right)}^{5}}$$

is applied so that the transport rate goes to zero when concentrations in the nephron exceed an upper limit. Without this factor, concentrations become unbounded when the flow in the collecting duct approaches zero. Physically, this is because the predicted concentration gradient increases as flow through the loop of Henle decreases. Without this factor the solution becomes mathematically unbounded when pressure drops low enough that all of the filtrate is reabsorbed because in this limit *q*_*a*_ and *dq*_*a*_/*dx* both approach zero, and the only way for *d*(*q*_*a*_*c*_*a*_)/*dx* to approach a constant value would be for *c*_*a*_ and/or its gradient to become unbounded. Since the concentration gradient drives fluid loss from the descending limb and the collecting duct, increases in the concentration gradient lead to further decreases in flow. The Hill coefficient of 5 in this multiplying factor is also arbitrarily assigned so that transport rapidly approaches zero when *c*_*a*_ exceeds *C*_*a,*max_. The value of the fixed parameter *C*_*a,*max_ set to 500 mM, so that the maximal Na^+^ concentration achieved at low flows is approximately 800 mM, associated with an approximately 5-fold magnification of the input concentration of *c*_*d*_(0) = 150 mM. (See below.) For pressures and flows that result in urine flows that are approximately equal to and greater than the baseline values, *c*_*a*_ remains well below *C*_*a,*max_ and the behavior of the model is not sensitive to the values of these fixed parameters.

Sodium reabsorption in the collecting duct is not explicitly accounted for in the model and the equation for Na^+^ flux in the collecting duct is

(18) $$\frac{d\left(q_{c}c_{c}\right)}{dx}=0.$$

This simplifying assumption is discussed below. Salt transport in the interstitial space combines active transport and passive permeation processes:

(19) $$\frac{d(q_{s}c_{s})}{dx}=+P(c_{a})-H_{d}(c_{s}-c_{d})-H_{c}(c_{s}-c_{c})\text{.}$$

Equation (19) assumes that the combined interstitial and vasa recta space gathers the sum of the fluxes from the other structures. Thus, as expressed by Keener and Sneyd,

(20) $$\frac{d(q_{s}c_{s})}{dx}=-\frac{d}{dx}(q_{d}c_{d}+q_{a}c_{a}+q_{c}c_{c})\text{.}$$

The boundary conditions for input into the descending limb assume that input concentration is equivalent to plasma sodium concentration of 150 mM and input flow is proportional to *Q*_*f*_, the glomerular filtration rate:

(21) $$\begin{array}{l} q_{d}(0)=\beta Q_{f},\\ c_{d}(0)=150\text{mM,} \end{array}$$

where *Q*_*f*_ = *Q*_*i*_ − *Q*_*e*_ is determined as a function of arterial pressure by the renal blood flow and filtration model component and *β* = 0.33 is a fixed constant accounting for reabsorption by the proximal tubule. Thus, the model assumes constant glomerulotubular balance and isotonic reabsorption of water and sodium from the proximal tubule [41].

The boundary conditions for the ascending limb are obtained from the assumption of continuity of concentration and flow at the turn of the loop of Henle:

(22) $$\begin{array}{l} q_{a}(D)=-q_{d}(D),\\ c_{a}(D)=c_{d}(D)\text{.} \end{array}$$

Similarly, the ascending limb feeds into the collecting duct

(23) $$\begin{array}{l} q_{c}(0)=-q_{a}(0),\\ c_{c}(0)=c_{a}(0)\text{.} \end{array}$$

The interstitial flow boundary condition is [15]

(24) $$\begin{array}{l} q_{s}(D)=0\\ q_{d}(0)+q_{s}(0)=q_{c}(D)\text{.} \end{array}$$

Equations (20), (21), (22), and (23) provide eight boundary conditions for the eight first-order differential equations described above. The eighth condition comes from conservation of total fluid flow [15], requiring that flow into the system at *x* = 0 equal flow out of the system at *x* = *D*.

Numerical discretization of the nephron model is described in the Appendix.

Since sodium reabsorption occurs in the model only in the ascending limb and the outflow of the ascending limb feeds directly into the collecting duct, the model does not explicitly account for sodium reabsorption in the distal tubule or the collecting duct. Thus all sodium reabsorption processes are represented by the ascending limb sodium transport rate *P*(*C*_*a*_). This simplifying approximation is justified by the fact that during formation of either concentrated or dilute urine, the majority of sodium reabsorption occurs via the ascending limb. Lumping all reabsorption processes into Equation (17) helps keep the model tractable and identifiable. Adding additional processes would add additional uncertainty in parameter values that would not be justified given the available data or yield any additional insight into the operation of the integrated model.

## Results

### **Model identification**

**Figure 2:**
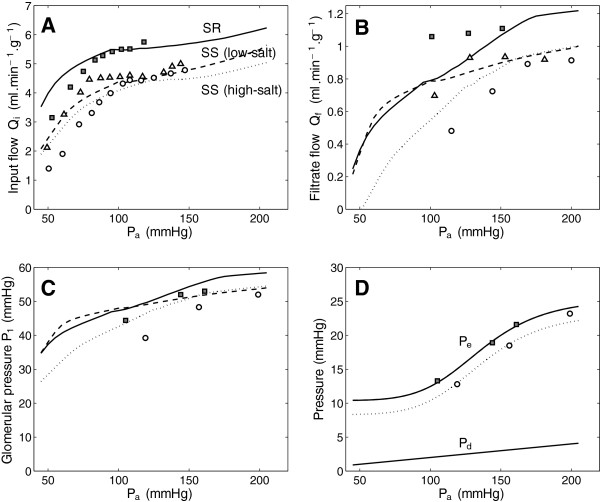
**Model predictions of renal blood flow and filtration compared to data from Dahl S (SS) rats on high-salt and low-salt diets and Dahl R (SR) rats on high-salt diets.** Data on renal flow, filtration rate, glomerular pressure, and pertibular efferent pressure are plotted as circles for SS high-salt fed animals, triangles for SS low-salt fed animals, and filled squares for SR animals. Model predictions based on the parameter values from Table 1 are compared to observed data. The difference between the high-salt and low-salt case is captured primarily by differences in afferent arterial resistance control parameters. Data in panel **A** are obtained from Figure 1 of Roman [12]; panel **B** from Figure five of Roman [12]; panel **C** and **D** from Figure six of Roman [12].

**Figure 3:**
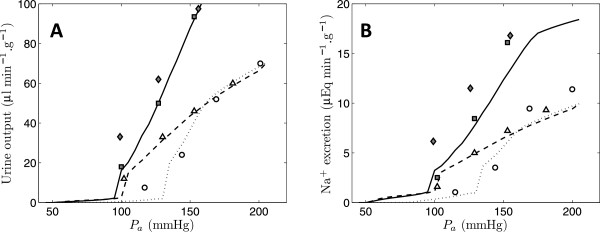
**Predicted pressure-natriuresis and pressure-diuresis relationships.** Urine output (*Q*_*u*_ = *Q*_*c*_(*x* = *D*)) and sodium excretion (*Q*_*c*_(*x* = *D*)· *C*_*c*_(*x* = *D*)) are plotted as functions of arterial pressure, for the Dahl salt-sensitive group on high-salt diet (circles) and low-salt diet (triangles) and for the Dahl salt-resistant group as filled symbols (squares and diamonds). Data for urine output and sodium excrection are obtained from Figure 3 of Roman [12] and Figure 5 of Roman and Kaldunski [13]. The data for the Dahl R group plotted as diamonds are obtained from Roman and Kaldunski [13]; all other data are obtained from Roman [12]. Model predictions for all cases use parameter values defined in Table 1.

Predictions of the renal flow and filtration model component are compared in Figure 2 to data on blood flow, filtration rate, glomerular pressure, and efferent pressure, as functions of arterial pressure in the SS (high-salt and low-salt) and SR (high-salt) rats. Predictions of urine output (*Q*_*u*_ = *Q*_*c*_(*x* = *D*)) and sodium excretion (*Q*_*c*_(*x* = *D*)· *C*_*c*_(*x* = *D*)) are compared in Figure 3 to data from SS (high-salt and low-salt) and SR (high-salt) rats as functions of arterial pressure. Data plotted in both Figures 2 and 3 used for model identification are obtained from Roman [12]. Additional independent data on urine output and sodium excretion in the SR rat were obtained from Roman and Kaldunski [13].

The 19 adjustable parameters invoked in this model are not identifiable for a given experimental group based on the six data sets (renal blood flow, filtration, glomerular pressure, efferent pressure, urine output, and sodium excretion as function of renal perfusion pressure) represented in these figures. However, the combined data set of pressures and flows versus arterial pressure for three experimental cases— prehypertensive (low-salt fed) and hypertensive (high-salt) SS rats and salt-resistant (high-salt) SR rats—provides independent data that can be compared to sixteen model-predicted functions of *P*_*a*_: *Q*_*i*_, *Q*_*f*_, *P*_*1*_, *P*_*e*_, *Q*_*u*_, and *Q*_*u*_· *C*_*c*_(*x* = *D*) under both high- and low-salt conditions for the SS and high-salt conditions for the SR. (Data on *P*_*1*_ and *P*_*e*_ as functions of *P*_*a*_ for the SS on low salt are not available; data for the SR were used to parameterize Equation (9) to represent *P*_*e*_(*P*_*a*_) for the SS on low salt.) If we assume that most model parameters attain the same values for all three groups, it is possible to determine identifiable parameter sets and to determine the minimal set of differences between the two conditions that is able to explain the observed data.

Specifically, if it is assumed that only two afferent arterial flow regulation parameters *Q*_*i*_^*o*^ and *R*_*a*_^0^ and the efferent arterial flow regulation parameters *m* and *R*_*e*_^max^ are different between the SR and SS (low-salt) cases and that only the parameters *R*_*a*_^0^, *R*_*e*_^max^, *m*, and *P*_*e0*_ are different between the SS low-salt and high-salt cases, then there are a total of 27 adjustable parameters that can be estimated by matching data to the 16 model-predicted functions in Figures 2 and 3. These parameters that are allowed to attain different values between the experimental groups govern how afferent arterial resistances are regulated in the model and do not directly affect glomerular filtration or transport in the nephron. Model simulations associated with the parameter values listed in Table 1 are plotted in Figures 2 and 3. The data on flows and pressures in SS and SR rats shown in Figure 2 are effectively captured by the model, with the exception of glomerular filtration rate in the SR rat. (The apparent mismatch between model prediction and reported data on glomerular filtration rate in the SR rat is discussed below.)

The predicted trends in afferent conductivity shown in Figure 1B may be compared to the measurements of Takenaka et al. [42], who observed that: (1.) afferent arterioles from low-salt SS animals maintain higher diameters at low pressures than those from high-salt animals; and (2.) arterioles from low-salt SS animals show a stronger constriction in response to increasing pressure than those from high-salt animals. These results are consistent with our model predictions. However, the observations of Takenaka et al., which made use of an isolated buffer perfused hydrophonetic kidney preparation, demonstrated total abolishment of the autoregulatory response in afferent arterioles in the high-salt case. An exact match between the model and the in vitro data of Takenaka et al. is not expected because the nature of the experiments of Takenaka et al. altered any sheer-dependent component of physiological diameter regulation and abolished tubular-glomerular feedback, and because the data of Roman show a clear, if blunted, autoregulation of renal blood flow and filtration in the high-salt animals.

The acute changes in sodium excretion and urine output in response to changes in renal perfusion pressure plotted in Figure 3 are termed the pressure-natriuresis and pressure-diuresis curves. These acute responses should not be confused with long-term relationships between pressure and sodium excretion and urine output, which are influenced by a number of hormonal, neural, and remodeling processes not accounted for here. Here, the acute pressure-natriuresis and diuresis phenomena are effectively reproduced by the model. Since the glomerular filtration and nephron transport parameters are held fixed for all experimental groups, the differences in afferent and efferent arteriole tone are responsible for greatly diminished rates of urine output and increased rate of sodium reabsorption in the SS (on high and low salt) compared to the SR.

**Figure 4:**
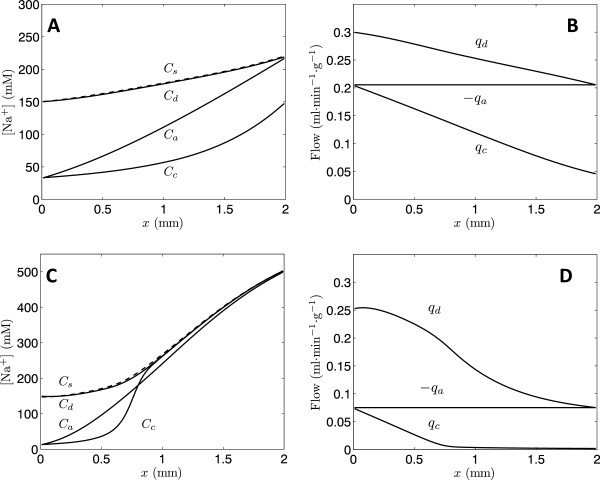
**Model-predicted sodium concentration and flow profiles in the nephron model.** Simulations are conducted using the parameter values for the Dahl R rat (Table 1). **A**. &**C**. Sodium concentrations as functions of distance along the nephron are plotted for the descending and ascending limb, collecting duct, and interstitium. **B**. &**D**. Flows as functions of distance along the nephron are plotted for the descending and ascending limb and collecting duct. The upper panel (A & B) reports model predictions for the baseline case with *q*_*d*_(0) = 0.300 ml·min^−1^·g^−1^ and *P*_*a*_ =125 mmHg. The lower panel (**C** &**D**) reports predictions for a lower pressure: *q*_*d*_(0) = 0.252 ml·min^−1^·g^−1^ and *P*_*a*_ = 95 mmHg. At the lower pressure the concentration gradient steepens and output flow drops to near zero.

Predictions of concentration and flow profiles in the nephron, based on the nephron model, are illustrated in Figure 4. The upper panel plots model predictions associated with an arterial pressure of 125 mmHg, near the baseline pressure of 126 ± 1 mmHg observed in the (high-salt) SR rats [12]. The lower panel plots model predictions associated with lowering the input pressure to 95 mmHg. Although the differences in input pressure and flow between the upper and lower panels are small, the predicted model behaviors show a major qualitative difference. The slightly lower input flow for the lower pressure simulation results in collecting duct flow that drops to near zero at the outlet at *x* = 2 mm. Also, at lower flow the concentration gradient is greater than at the higher flow. At arterial pressures 95 mmHg and below, the maximal concentrations at *x* = 2 mm are approximately 500 mM, over a three-fold increase of the input concentration of 150 mM.

To summarize the findings of comparing model predictions to data from Roman [12] on high-salt SR and hypertensive (high-salt) and prehypertensive (low-salt) SS rats, the observed differences in renal function may be explained primarily by differences in the control of afferent and efferent resistance and in sodium reabsorption kinetics.

## Discussion

### **Mechanisms of pressure-natriuresis and pressure-diuresis**

Using a simple mathematical model to simulate blood flow regulation, glomerular filtration, and medullary solute transport in the kidney, we have analyzed data from Dahl S and Dahl R rats to investigate the potential mechanistic underpinnings of renal function observed in these animals. While the pressure-natriuresis and pressure-diuresis relationships illustrated in Figure 3 have long been recognized as playing a central role in the long-term control of blood pressure [43], the biophysical mechanisms underlying these phenomena have not been fully resolved. One school of thought maintains that because renal blood flow and glomerular filtration rate do not change over a wide range of arterial pressure, the observed decrease in sodium and water reabsorption associated with an increase in pressure could not be substantially impacted by an increase in the rate of filtrate delivery to nephrons. Observations of reduced sodium-hydrogen exchanger activity in the proximal tubule [44] and increased medullary blood flow [45,46] in response to acute and chronic increases in blood flow point to mechanisms that reduce sodium reabsorption. Another widely held view is that both acute and chronic increases in arterial pressure increase filtration rate and thus directly increase sodium and water excretion through simple mechanical transduction. In a textbook explanation of the pressure-diuresis phenomenon, this primary mechanical response is enhanced by other contributing mechanisms, including the renin-angiotensin system, changes in medullary blood flow, regulation of proximal tubule sodium transport [41].

Our model simulations, as well as the data analyzed here, are consistent with the pressure-natriuresis and pressure-diuresis phenomena emerging from the mechanical relationships between renal pressure, flow, and filtration. Specifically, in the model increasing arterial pressure causes increased glomerular pressure, which causes increasing filtration rate. For the SS rat data sets, an increase in glomerular pressure of 20–30% over the observed pressure range results in an increase in filtration rate of 30% in the low-salt case and almost 90% in the high-salt case. When pressure increases from 100 to 180 mmHg, filtrate flow increases from 700 to over 900 μl·min^−1^·g^−1^ while urine output increases from 10 to 60 μl·min^−1^·g^−1^ in low-salt case. In the high salt case, filtrate flow increases from approximately 480 to 900 μl·min^−1^ while urine output increases from 6 to 68 μl·min^−1^·g^−1^ over the pressure range of 120 to 200 mmHg. Thus the slope of filtrate flow (*Q*_*f*_) versus arterial pressure can be substantially steeper than the slope of urine output (*Q*_*u*_) versus arterial pressure, even over the pressure range for which blood flow is autoregulated. For these cases the relative change in urine output over the pressure range is much greater than the relative change in filtrate flow because at the lowest pressures nearly all of the filtrate is reabsorbed.

In contrast, the SR data show relatively little increase in filtration over the observed pressure range for the three data points in Figure 2B. For this case, an increase in filtration of approximately 50 μl·min^−1^·g^−1^ is associated with an increase of 65–80 μl·min^−1^·g^−1^ in urine output. For this case the model is not able to capture the nearly constant *Q*_*f*_ as a function of *P*_*a*_ because the glomerular pressure is observed to increase from 44 to 53 mmHg over the same arterial pressure range. Recall that the driving force for filtration is hydrostatic pressure difference minus the oncotic pressure of approximately 28 mmHg. Since the 8 mmHg increase in glomerular pressure over the observed range of arterial pressure represents an approximately 30% increase in driving force for filtration, the model tends to under-represent the slope of *P*_*1*_ versus *P*_*a*_ while over representing the slope of *Q*_*f*_ versus *P*_*a*_. It is unclear how to resolve the substantial differences in driving force for filtration with the apparently constant filtration rate observed in the SR rat. The model predicts that *Q*_*f*_ increases roughly 20% over the observed 50 mmHg range of arterial pressure, while measurements in the SS rat and in other rat strains and other species show increases of anywhere from 10% to greater than 20% over the pressure range of autoregulated blood flow [47-49].

**Figure 5:**
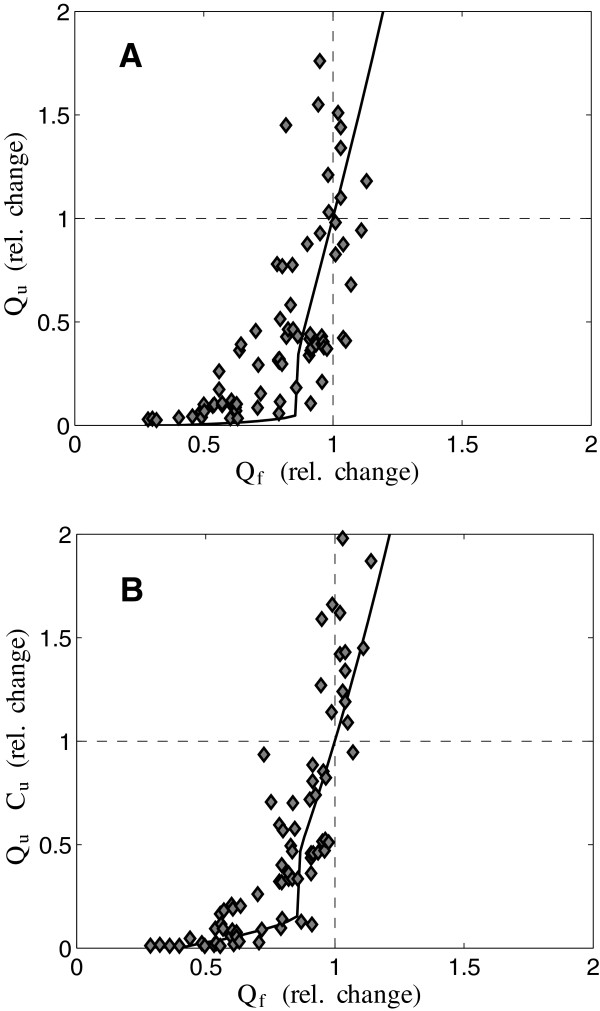
**Relationship between urine output and sodium excretion and glomerular filtration rate. A**. Relative change in urine output *Q*_*u*_ is plotted versus relative change in filtration *Q*_*f*_ over the arterial pressure range studied in Figure 2. **B**. Relative change in urine output *Q*_*u*_ is plotted versus relative change in filtration *Q*_*f*_ . Data from Thompson and Pitts [14] from normal dogs are plotted for comparison. All calculations are for the Dahl R rat parameter set.

The relationship between sodium excretion and glomerular filtrate rate is further explored in Figure 5 by comparing the model predictions of these variables to the data of Thompson and Pitts [14]. Here, model predictions and data are plotted as percent of control since the data are obtained from dog and the model is parameterized for the SR rat. Note that this comparison represents a model prediction where no parameter adjustment has been done to match the data. The nonlinear nature of the relationship is effectively captured by the model, where relatively small increases in filtration rate can effect relatively large changes in sodium excretion. Furthermore, the simplified model reveals the extent to which mechanisms not included in the model may be important contributors to the physiological phenomena explored. Specifically, Figure 5 shows that a 20% increase in filtration rate above baseline level elicits a 100% increase in model-predicted urine output rate, for the SR parameter set.

**Figure 6:**
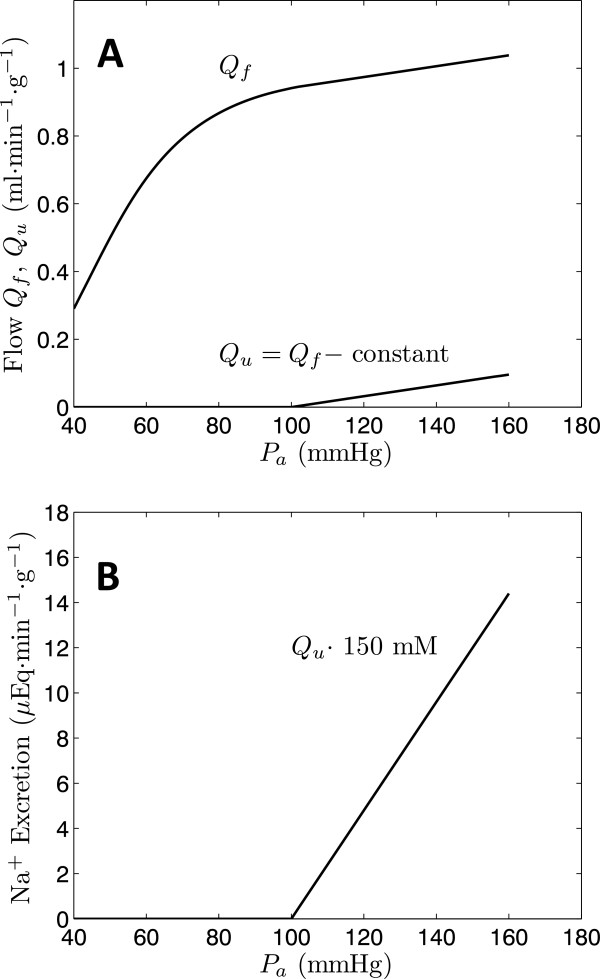
**Conceptual model for pressure-diuresis and pressure-natriuresis.** Idealized curves are used to illustrate the hypothesized relationships between glomerular filtration flow (*Q*_*f*_), urine output (*Q*_*u*_), and sodium excretion following acute changes in renal arterial pressure (*P*_*a*_).

The mechanistic explanation for the pressure-diuresis and pressure-natriuresis phenomena that emerges is illustrated in Figure 6. The upper panel plots conceptualized curves representing glomerular filtration flow and urine flow as functions of arterial pressure. Consistent with the available data, the slopes of glomerular filtration flow and urine flow versus pressure are of the same order of magnitude over the autoregulated range, here taken to be *P*_*a*_ = 100 to 160 mmHg. If the slopes are the same over this pressure range, then *Q*_*u*_ can be approximated as *Q*_*f*_ minus a constant reabsorbed volume. Assuming that sodium concentration remains approximately constant at arterial pressure above the baseline 100 mmHg, the pressure-diuresis relationship of the bottom panel is obtained. While this conceptual model is highly simplified, it does effectively illustrate the basic mechanism that emerges from our mathematical model: since glomerular filtration flow is much larger than urine flow, a relatively small increase in glomerular filtration can cause a relatively large increase in urine output. Thus, this explanation requires that glomerular filtration does increase, albeit slightly, as renal arterial pressure is acutely increased. If, as has been hypothesized, glomerular filtration remains exactly constant as arterial pressure is acutely increased, then this mechanism cannot explain the observed pressure-diuresis and pressure-natriuresis relationships.

The data from Thompson and Pitts, as well as the data of Roman analyzed in Figures 2 and 3, indicate that the slope of *Q*_*f*_ versus *P*_*a*_ may be lower in normal animals than that captured by the model and that additional secondary mechanisms may be necessary to satisfactorily explain the pressure-diuresis/natriuresis phenomenon. Clearly, mechanical transduction is not the only mechanism at work in vivo. Yet even without a model simulation, it is apparent from the raw data that a given change in pressure can induce a greater change in glomerular filtration than in urine output over the pressure range for which blood flow is autoregulated. The model reveals the extent to which the relationship between acute changes in arterial pressure and glomerular filtration can explain the observed pressure-natriuresis and pressure-diuresis relationships. These findings highlight the direct effects of pressure on influencing urine production by delivering increased filtrate to the proximal tubule. The ability of the model to match observed relationships between arterial pressure and glomerular pressure, urine flow, and sodium excretion depends on the predicted increase in glomerular filtration with pressure that is not apparent in the SR data set. For the model to capture the phenomenon of constant glomerular filtration over the arterial pressure range of 100 to 150 mmHg observed in the SR group would require the introduction of some (unknown) mechanism that reduces glomerular hydraulic permeability in response to acute increases in pressure. Furthermore, since it does not account for hormonal or nervous factors, or changes in medullary blood flow and transporter activities, the model reveals that these factors are not necessary to explain the acute pressure-natriuresis and pressure-diuresis phenomena, at least in the SS rat on low- and high-salt diets.

### **Physiological differences between SS and SR groups**

In addition to revealing insight into how sodium excretion and urine output are influenced by perfusion pressure, model analysis reveals potential mechanistic underpinnings of differences in renal function observed in SR and SS rats when fed on low-salt versus high-salt diets. Our strategy identifies the minimal differences between model parameterizations necessary to explain the data from these groups. Specifically, it was found that differences in five parameters associated with blood flow control (see Table 1) are able to explain a host of differences in renal function observed among the three groups (see Figures 2 and 3).

In developing the model presented here and determining the difference in parameter values necessary to explain the groups, the goal is not to capture all relevant physiological processes impacting renal function and blood pressure regulation in the rat. Indeed several mechanisms important to the renal response to changes in blood pressure, including changes in proximal tubule sodium transport [44] and inner medullary blood flow [45], are not accounted for. In contrast, by focusing on a well-defined set of identifiable physiological processes, we are able to determine a minimal set of processes to explain the data and what differences in those processes are necessary to explain the different experimental cases.

The different parameterizations used to explain the different experimental groups point to increases in afferent resistance and decreases in sodium transport rate as one moves from lower-pressure to higher-pressure animals. The increase in afferent resistance is able to explain all of the qualitative differences between observed data on renal function—lower flows, filtration rates, and glomerular pressure as well as the shift in the pressure-natriuresis and pressure-diuresis relationships in higher pressure animals. (Since the data analyzed here are obtained from denervated kidneys, the predicted differences in afferent arterial tone cannot be explained based on differences in sympathetic tone, unless chronic differences in sympathetic tone had the effect of chronically altering afferent arterial tone in a way that is reflected in denervated kidneys.)

This observed shift (compared to lower pressure controls) of the pressure-natriuresis relationship to higher pressure necessarily occurs in hypertension. This is because net sodium balance, by definition, must occur at a higher pressure in hypertension than in normotension. The view that the chronic pressure-natriuresis relationship (also called the renal function curve) observed in normal animals is effectively infinitely steep implies that the kidney can maintain blood volume and sodium at nearly constant levels in response to small changes in pressure associated with salt-loading and volume expansion [50]. Furthermore, the steepness of the renal function curve forms the basis of the theory that chronic hypertension in angiotensin- and salt-induced models is caused by renal dysfunction leading to decreased sodium excretion at a given arterial pressure [1-6].

While it is debated whether and/or when renal dysfunction represents the primary cause of chronic hypertension in the SS rat (and other animal models) [51,52], it is clear that the high-salt diet does cause a shift in the acutely measured pressure-natriuresis relationship in the SS rat, as illustrated in Figure 3. These changes are shown here to be underpinned by changes in renal afferent and efferent arterial resistance. If indeed a shift in the pressure-natriuresis relationship is the primary cause of elevated arterial pressure in the Dahl S rat, then alternation in how renal afferent and efferent arterial resistances are regulated represents the primary cause of chronic hypertension in the Dahl S rat.

### **Assumptions and simplifications of the model**

As discussed in the methods section, the developed model for blood flow, glomerular filtration, and sodium transport in the proximal tubule, nephron, and collecting duct developed here is relatively unsophisticated compared to a number of previously developed models of the three-dimensional architecture of the tubules and vasculature [16-19], models of renal flow regulation and tubuloglomerular feedback [20-22], transport in the proximal tubule and cortex [23-25], medulla [17,18,26-35], collecting duct [34,36], and other components [16]. Nor does the model account for different roles for the inner and outer medulla, in terms of tubular function or blood flow, or explicitly for the transport of urea or other solutes. Yet despite the simplifications, the model represents the only available computational model of whole-kidney function that integrates blood flow regulation, glomerular filtration, distributed solute and volume fluxes along a nephron, and tubuloglomerular feedback, to compare whole-kidney pressure-natriuresis and pressure-diuresis phenomena to experimentally observed data.

To a certain degree the level of simplification adopted by the model is justified by the nature of the data analyzed here and the specific questions addressed by the model analysis. The appropriate level of complexity represented by a model is the lowest (most simple) that can capture the biophysical processes underlying the phenomena studied. Based on this standard, the present model may be judged as a reasonable, if imperfect, simplification. The most obvious feature that the model does not capture well is the phenomenon of nearly constant filtration rate observed in the SR group. It is not known what anatomical and physiological features not represented in the current model are critical to improve the behavior of the model in comparison to this observation. While the model makes a number of simplifying assumptions, it is not clear that relaxing any one of those simplifying assumptions would explain the apparent disconnect between driving force for filtration and filtration rate in the SR group. What is clear is that the basic model introduced here represents a useful framework for exploring such questions in a systematic matter.

## Conclusions

Analysis of data on renal blood flow, filtration, pressure-diuresis and pressure-natriuresis phenomena in Dahl S and Dahl R rats using a simple mathematical model reveals a hypothetical mechanistic explanation for the observed pressure-diuresis and pressure-natriuresis relationships. Idealized curves plotted in Figure 6 illustrate the hypothesized relationships between glomerular filtration flow (*Q*_*f*_), urine output (*Q*_*u*_), and sodium excretion following acute changes in renal arterial pressure (*P*_*a*_). Increasing pressure is associated with a relatively small increase in glomerular filtration, which increases delivery of filtrate to the nephron, leading to increased urine production. This simplified conceptual model requires that glomerular filtration increases slightly as renal arterial pressure is acutely increased. Furthermore, differences between Dahl salt-sensitive (SS) and salt-resistant (SR) rats in renal filtration and urine production are explained in terms of difference in blood flow regulation.

## Appendix: Discretization of nephron equations

Equations (12), (13), and (14) are discretized using finite differences

(25) $$\begin{array}{l} Q_{d}^{<i>}=Q_{d}^{0}+\Delta xK_{d}\sum_{j=1}^{i}(-\Delta P_{d}+2RT(C_{d}^{<j>}-C_{s}^{<j>}))\\ Q_{a}=-Q_{d}^{<N>}\\ Q_{c}^{<i>}=Q_{d}^{<N>}+\Delta xK_{c}\sum_{j=1}^{i}(-\Delta P_{c}+2RT(C_{c}^{<j>}-C_{s}^{<j>}))\\ Q_{s}^{<i>}=Q_{c}^{<N>}-(Q_{d}^{<i>}+Q_{a}^{<i>}+Q_{c}^{<i>}) \end{array}$$

where *i* = 1, 2, …, *N* is the element index and *Q*_*d*_^0^ = *q*_*d*_(0) is the input flow into the descending limb. The discrete variables *Q*_*d*_^<*i*>^, *Q*_*a*_^<*i*>^, *Q*_*c*_^<*i*>^, and *Q*_*s*_^<*i*>^ are numerical approximations for the continuous variables *q*_*d*_(*x*), *q*_*a*_(*x*), *q*_*c*_(*x*), and *q*_*s*_(*x*). The equation for *Q*_*s*_^<*i*>^ is based on mass conservation and the boundary condition *Q*_*s*_^<*N*>^ = 0.

The concentrations satisfy numerical approximations of Equations (16) for the descending limb:

(26) $$\begin{array}{l} Q_{d}^{<i>}C_{d}^{<i>}-Q_{d}^{0}C_{d}^{0}=\Delta xH_{d}(C_{s}^{<i>}-C_{d}^{<i>}),i=1\\ Q_{d}^{<i>}C_{d}^{<i>}-Q_{d}^{<i-1>}C_{d}^{<i-1>}\\ \;=\Delta xH_{d}(C_{s}^{<i>}-C_{d}^{<i>}),i=2,3,\ldots ,N \end{array}$$

Equation (17) for the ascending limb:

(27) $$\begin{array}{l} C_{a}^{<i>}=C_{d}^{<N>}+\frac{\Delta x}{Q_{a}}P(C_{a}^{<i>}),i=N\\ C_{a}^{<i>}=C_{a}^{<i+1>}+\frac{\Delta x}{Q_{a}}P(C_{a}^{<i>}),i=1,2,\ldots ,N-1 \end{array}$$

Equation (18) or the collecting duct:

(28) $$\begin{array}{l} Q_{c}^{<i>}C_{c}^{<i>}-(-Q_{a})C_{a}^{<1>}=\Delta xH_{c}(C_{s}^{<i>}-C_{c}^{<i>}),i=1\\ Q_{c}^{<i>}C_{c}^{<i>}-Q_{c}^{<i-1>}C_{c}^{<i-1>}\\ \;=\Delta xH_{c}(C_{s}^{<i>}-C_{c}^{<i>}),i=2,3,\ldots ,N \end{array}$$

and Equation (18) for the interstitium:

(29) $$\begin{array}{l} Q_{s}^{0}=Q_{s}^{<1>}-\left[Q_{d}^{0}-(Q_{d}^{<1>}+Q_{a}+Q_{c}^{<1>})\right]\\ Q_{s}^{<i>}C_{s}^{<i+1>}-Q_{s}^{0}C_{s}^{<i>}=\Delta xH_{c}(C_{c}^{<i>}-C_{s}^{<i>})\\ \;+\Delta xH_{d}(C_{d}^{<i>}-C_{s}^{<i>})+\Delta xP(C_{a}^{<i>}),i=1\\ Q_{s}^{<i>}C_{s}^{<i+1>}-Q_{s}^{<i-1>}C_{s}^{<i>}=\Delta xH_{c}(C_{c}^{<i>}-C_{s}^{<i>})\\ \;+\Delta xH_{d}(C_{d}^{<i>}-C_{s}^{<i>})+\Delta xP(C_{a}^{<i>}),i=2,3,\ldots ,N-1\\ Q_{s}^{<i>}C_{s}^{D}-Q_{s}^{<i-1>}C_{s}^{<i>}=\Delta xH_{c}(C_{c}^{<i>}-C_{s}^{<i>})\\ \;+\Delta xH_{d}(C_{d}^{<i>}-C_{s}^{<i>})+\Delta xP(C_{a}^{<i>}),i=N \end{array}$$

The input concentration and flow are obtained from the boundary conditions *Q*_*d*_^0^ = *q*_*d*_(0) and *C*_*d*_^0^ = *c*_*d*_(0). Qs^<*N*>^ and *C*_*s*_^*D*^ are the input vasa recta flow and concentration (at *x* = *D*); *Q*_*s*_^<*N*>^ = *q*_*s*_(*D*) = 0. As long as *Q*_*s*_^<*N*>^ = 0, the value of *C*_*s*_^*D*^ is arbitrary.

These equations are solved using an iterative method. Computer codes for the model can be obtained by contacting the author.

## Authors’ contributions

DAB and MM developed the model, carried out the simulation studies, and drafted the manuscript. All authors read and approved the final manuscript.
